# Modeling the β-secretase cleavage site and humanizing amyloid-beta precursor protein in rat and mouse to study Alzheimer’s disease

**DOI:** 10.1186/s13024-020-00399-z

**Published:** 2020-10-19

**Authors:** Lutgarde Serneels, Dries T’Syen, Laura Perez-Benito, Tom Theys, Matthew G. Holt, Bart De Strooper

**Affiliations:** 1 Centre for Brain and Disease Research, Flanders Institute for Biotechnology (VIB), Leuven, Belgium; 2 Department of Neurosciences and Leuven Brain Institute, KU Leuven, Leuven, Belgium; 3 Computational Chemistry, Janssen Research & Development, Janssen Pharmaceutica NV, Beerse, Belgium; 4 Department of Neurosciences, Research Group Experimental Neurosurgery and Neuroanatomy, KU Leuven, Leuven, Belgium; 5 UK Dementia Research Institute at UCL, University College London, London, UK

**Keywords:** Knock-in, Rodent animal models, Alzheimer’s disease, Amyloid-beta precursor protein, Presenilin

## Abstract

**Background:**

Three amino acid differences between rodent and human APP affect medically important features, including β-secretase cleavage of APP and Aβ peptide aggregation (De Strooper et al., EMBO J 14:4932-38, 1995; Ueno et al., Biochemistry 53:7523-30, 2014; Bush, 2003, Trends Neurosci 26:207–14). Most rodent models for Alzheimer’s disease (AD) are, therefore, based on the human *APP* sequence, expressed from artificial mini-genes randomly inserted in the rodent genome. While these models mimic rather well various biochemical aspects of the disease, such as Aβ-aggregation, they are also prone to overexpression artifacts and to complex phenotypical alterations, due to genes affected in or close to the insertion site(s) of the mini-genes (Sasaguri et al., EMBO J 36:2473-87, 2017; Goodwin et al., Genome Res 29:494-505, 2019). Knock-in strategies which introduce clinical mutants in a humanized endogenous rodent *APP* sequence (Saito et al., Nat Neurosci 17:661-3, 2014) represent useful improvements, but need to be compared with appropriate humanized wildtype (WT) mice.

**Methods:**

Computational modelling of the human β-CTF bound to BACE1 was used to study the differential processing of rodent and human APP. We humanized the three pivotal residues we identified G676R, F681Y and R684H (labeled according to the human APP770 isoform) in the mouse and rat genomes using a CRISPR-Cas9 approach. These new models, termed mouse and rat App^hu/hu^, express APP from the endogenous promotor. We also introduced the early-onset familial Alzheimer’s disease (FAD) mutation M139T into the endogenous Rat *Psen*1 gene.

**Results:**

We show that introducing these three amino acid substitutions into the rodent sequence lowers the affinity of the APP substrate for BACE1 cleavage. The effect on β-secretase processing was confirmed as both humanized rodent models produce three times more (human) Aβ compared to the original WT strain. These models represent suitable controls, or starting points, for studying the effect of transgenes or knock-in mutations on APP processing (Saito et al., Nat Neurosci 17:661-3, 2014). We introduced the early-onset familial Alzheimer’s disease (FAD) mutation M139T into the endogenous Rat *Psen*1 gene and provide an initial characterization of Aβ processing in this novel rat AD model.

**Conclusion:**

The different humanized APP models (rat and mouse) expressing human Aβ and PSEN1 M139T are valuable controls to study APP processing in vivo allowing the use of a human Aβ ELISA which is more sensitive than the equivalent system for rodents. These animals will be made available to the research community.

## Background

Alterations in Aβ generation and aggregation are central to AD [1–5]. More than 177 transgenic models overexpressing mutated human APP and/or PSEN are currently available [6]. While these models lack tangles and do not develop symptoms of dementia, they remain good models in which to investigate mechanisms of amyloid accumulation. However, these models do have some drawbacks, including the potential artefacts directly caused by APP overexpression (for instance dysregulation of GABAergic neurotransmission [7]) and the random integration of transgenes (which could impact on the correct expression of adjacent genes) [8–10]. Recent improvements in genome editing now make it relatively straightforward to introduce subtle disease-causing mutations, or to humanize genes, rather than overexpressing mutant mini-genes [11].

APP knock-in models are available that express clinical AD mutations (KM 670/671NL, E693G, I716F) in the endogenous mouse sequence [12, 13]. These models develop amyloid pathology and interesting phenotypes without potential artefacts introduced by APP overexpression. However, the WT control (humanized APP without these mutations) is not readily available. The three amino acid substitutions (G676R, F681Y and R684H) needed to humanize the mouse Aβ sequence have a profound effect on Aβ generation [14].

In the current study we show, using in silico modelling, how these substitutions affect the interaction with BACE1. We have also used CRISPR-Cas9 technology to scarlessly humanize the endogenous Aβ sequence in the mouse and rat *App* genes [15] and investigate the effects on APP processing. We also created a PSEN1 knock-in mutation to generate a rat model for AD.

## Material and methods

### Mice

Mice *App*^em1Bdes^ with a humanized Aβ sequence (G676R, F681Y, R684H) were generated using CRISPR-Cas9 technology to target exon 16 of the mouse *App* gene. RNA guides were selected using the CRISPOR web tool. Guide 5′-GCAGAAUUCGGACAUGAUUC-3′ and 5′-GUCCGCCAUCAAAAACUGGU-3′ were selected and tested in mouse embryonic fibroblast (MEF) cells for cleaving efficiency. To promote homologous recombination directed repair [16] we made use of a ssODN repair template to mutate the target amino acids and to introduce two silent nucleotide substitutions. The first silent substitution destroys an EcoRI restriction cleaving site, facilitating genotyping. The second silent substitution prevents Cas9 cleaving the modified locus. Ribonucleoproteins (RNPs) containing 0.3 μM purified Cas9HiFi protein (Integrated DNA Technologies, IDT), 0.6 μM CRISPR RNAcrRNA, 0.6 μM trans activating crRNA (IDT) and 10 ng/μl ssODN (5′-tactttgtgtttgacgcagGTTCTGGGCTGACAAACATCAAGACGGAAGAGATCTCGGAAGTGAAGATGGATGCAGAATTtaGACATGATTCAGGATaTGAAGTCCaCCATCAgAAACTGgtaggcaaaaataaactgcctctccccgagattgcgtctggccagatgaaatacgtggcacctcgtggcttgtcctgtgt-3′) were injected into the pronucleus of 72 C57Bl6J embryos by microinjection in the Mouse Expertise Unit of KU Leuven. One positive pup was identified by PCR and restriction analysis. Sanger sequencing of *App* exon 16 region, as well as the 5 most likely off target sites predicted by the CRISPOR web tool, confirmed correct targeting (Additional file 1) and absence of spurious events at other sites. The founder mouse was backcrossed over two generations using C57BL6J mice before a homozygous colony was established, which was designated App^hu/hu^. The strain is maintained on the original C57Bl6J background by backcrossing every 5th generation. Standard genotyping is performed by PCR with primers 5′-taggtggtggttaatggtt-3′ and 5′-cgtagctgcaacgttggact-3′ followed by digestion of the PCR product with EcoRI.

App^tm3.1Tcs^ [12] also known as App NL-G-F and Tg (Thy1-MAPT)^22Schd^ [17] also known as Thy-Tau22 mice were used as positive controls during histological examination. Mice are kept on a C57Bl6J background and both females and males were included in the study. Mice are housed in cages enriched with wood wool and shavings as bedding, and given access to water and food ad libitum. All experiments were approved by the Ethical Committee for Animal Experimentation at the University of Leuven (KU Leuven).

### Rats

As the rat is one of the most studied model organisms [18], and until recently no knock-in rat models of AD were available [19], we set out to humanize the Aβ sequence in rats using a similar strategy as we used in the mouse. Two gRNAs (Additional file 1), 5′-GUGAAGAUGGAUGCGGAGUU-3′ and 5′-UUUUGCAUACCAGUUUUUGA-3′, Cas9 mRNA and an oligo donor with targeting sequence (flanked by 120 bp homologous sequences on both sides) were co-injected into zygotes of Long Evans rats We also introduced the early-onset familial Alzheimer’s disease (FAD) mutation M139T [20] into the endogenous Rat *Psen*1 gene. To target *Psen*1. Cas9 mRNA, sgRNA 5′-GAUGACACUGAUCAUGAUGG-3′ and an oligo donor containing the ATG/ACC substitution with 120 bp homologous sequences were co-injected into zygotes (Additional file 2). F0 rats were genotyped after weaning using PCR and Sanger sequencing. Founder rats carrying the humanized Aβ sequence and M139T mutant allele were crossed twice with WT Long Evans rats (Charles River). Rats homozygous for the humanized APP KI were obtained after crossing heterozygous offspring. A breeding colony homozygous for the humanized Aβ sequence and heterozygous for the *Psen*1M139T allele was established and designated *App*^hu/hu^;*Psen*1M139T. Standard genotyping for the APP KI mutation is done with the forward primer 5′-caTGATTCAGGCTaCGAAGTCCat-3′ and a common reverse primer 5′-CTCAGTGGTAATACGCCTGCCTAGC-3′. 5′-TGATTCAGGCTtCGAAGTCCgc-3′ is the forward primer for amplification of the wildtype *App* allele. For *Psen*1, genotyping is performed by PCR with a WT specific forward primer 5′-cgatcttgaatgccgccatcatg − 3′, or a M139T specific forward primer 5′-cgatcttgaatgccgccatcacc-3′, together with a common reverse primer 5′-ctgcacatgtacactctggcaag-3′. Rats are kept on a Long Evans background and backcrossed every fifth generation to WT rats. Both females and males were included in the study. Rats are housed in cages enriched with wood wool and shavings as bedding, and given access to water and food ad libitum. All experiments were approved by the Ethical Committee for Animal Experimentation at the University of Leuven (KU Leuven).

### Human tissue samples

Human brain samples were resected from the lateral temporal neocortex and were obtained from patients who underwent amygdalohippocampectomy for medial temporal lobe seizures. Samples were collected at the time of surgery and immediately transferred to the laboratory for processing. All procedures were conducted according to protocols approved by the local Ethical Committee of KU Leuven (protocol number S61186).

### Sample collection and protein analysis

Three female and 3 male mice and rats of the indicated genotypes were aged to 14 weeks and then euthanized by carbon dioxide overdose, followed by intracardial perfusion of ice-cold phosphate buffered saline. Brains were removed from the skull, and the cerebrum was snap frozen using liquid nitrogen and stored at − 70 °C until further processing. Half a brain hemisphere was weighed and homogenized in a bead mill using 5 volumes of buffer containing 20 mM Tris, 250 mM sucrose, 0.5 mM EDTA,0.5 mM EGTA (pH 7.4 HCl) supplemented with cOmplete™ protease inhibitor cocktail (Roche) and PhosSTOP™ (Sigma). This homogenate was divided in three fractions of 250 μl. One fraction was used to extract soluble Aβ using 0.4% Diethylamine treatment for 30 min at 4 °C. Following high speed clearing at 100,000 g for 1 h (at 4 °C), the sample was neutralized by adding 1/10 volume of 0.5 M Tris-HCl (pH 6.8) and analyzed by ELISA. To obtain the guanidine HCl (GuHCl)-soluble fraction, the 100,000 g pellet was washed with 0.4% Diethylamine before solubilization in 6 M GuHCl, 50 mM Tris-HCl (pH 7.6), supplemented with cOmplete™ protease inhibitor cocktail (Roche) and PhosSTOP™ (Sigma), and sonication using a micro-tip for 30 s at 10% amplitude (Branson). After incubation for 1 h at 25 °C with agitation (600 rpm), the sample was cleared by spinning at 100,000 g, diluted to 0.1 M GuHCl and analyzed by ELISA. Aβ38, Aβ40 and Aβ42 levels were quantified on Meso Scale Discovery (MSD) 96-well plates using ELISA and antibodies provided by Dr. Marc Mercken (Janssen Pharmaceutica). Monoclonal antibodies JRFcAβ38/5, JRFcAβ40/28 and JRFcAβ42/26, which recognize the C terminus of Aβ species terminating at amino acid 38, 40 or 42, respectively, were used as capture antibodies. JRF/rAβ/2 (rodent specific antibody) or JRFAβN/25 (human specific antibody) labeled with sulfo-TAG were used as the detection antibodies. Human Aβ43 was measured using the amyloid-beta (1–43) high sensitivity ELISA kit from IBL. To measure APP protein in the brain samples, 250 μl of homogenate were supplemented with 1% Triton X100, incubated for 30 min on ice and cleared for 30 min at 14,000 g (at 4 °C). For the extraction of soluble MAPT protein, 250 μl of a buffer containing 300 mM NaCl, 50 mM Tris-HCl, 150 mM NaCl, 1 mM EDTA, 2% NP-40, 0.5% sodium deoxycholate (pH 7.5), supplemented with cOmplete™ protease inhibitor cocktail (Roche) and PhosStop™ (Sigma), was added to 250 μl of brain homogenate. The sample was sonicated and incubated for 30 min on ice and cleared by centrifugation at 14,000 g (at 4 °C) for 30 min. When indicated, cell lysates were dephosphorylated after dialysis against 50 mM Tris-HCl (pH 7,6) using Calf Intestine Phosphatase (Bioke). Total protein content of the cell lysates was measured using a Biorad protein assay kit. Fifty micrograms of protein were loaded in reducing and denaturing conditions on NuPAGE™ (Thermo) gels and subjected to electrophoresis. Following separation, proteins were transferred to nitrocellulose membrane for western blotting. Membranes were blocked with 5% non-fat milk Tris buffered saline, containing 0.1% Tween 20, and incubated with the indicated primary antibodies, washed, and incubated with horseradish peroxidase conjugated secondary antibodies (Biorad). Blots were developed using the ECL Renaissance kit (Perkin Elmer). Primary antibodies used in this study were B63 (against the C-terminal amino acids of APP (previous used in [21], 1/1000)), 82E1 (IBL, 1/500), JRF/rAβ/2 (Janssen, 1/1000), anti-human TAU (Dako, 1/1000), anti-3RTAU (Millipore, 1/1000), anti-4RTAU (CosmoBio, 1/1000) and Anti-Actb clone AC-15 (Sigma, 1/20,000). Intensities of the bands were quantified with Aida/2D densitometry software.

All data are presented as mean ± SD, and were analyzed by GraphPad Prism 8. Unpaired two way Student’s t test and one-way ANOVA were used for group comparisons. *P* < 0.05 was considered statistically significant.

### Histology

Rats and control mice were euthanized with an overdose of carbon dioxide and transcardially perfused with PBS. Brain tissue was subsequently harvested and post-fixed overnight in 4% PFA in PBS. Brains were cut in serial sections of 40 μm thickness with a vibrating microtome (Leica). For each sample, six series of sections were sequentially collected in free-floating conditions and permeabilized for 30 min with PBST (0.2% Triton X-100 in PBS) and blocked for 2 h with 5% normal donkey serum in PBST. Antigen retrieval was performed by boiling the sections for 1 min in 10 mM sodium citrate (pH 6) in a microwave. After three rinses with 0.1% Tween 20 in PBS, sections were incubated for 20 min with 10 μM X34 (Sigma) in 0.1% NaOH made in 40% Ethanol washed and incubated overnight at 4 °C with primary antibodies against Aβ: 82E1 (IBL, 1/150) or 6E10 (BioLegend, 1/200). The antibody stained sections were washed three times with 0.2% TritonX100-PBS and incubated with Alexa594 Donkey anti-mouse IgG (Thermo Fisher, 1/300) for 2 h at RT. After final washes with 0.2% TritonX100-PBS, sections were counterstained with TO-PRO (Thermo Fisher, 1/1000) and after three final washes were mounted on super frost microscope slides. Sections were visualized on a Nikon A1R Eclipse confocal system.

### Computational model of the humanized β-CTF bound to BACE1

**Fig. 1:**
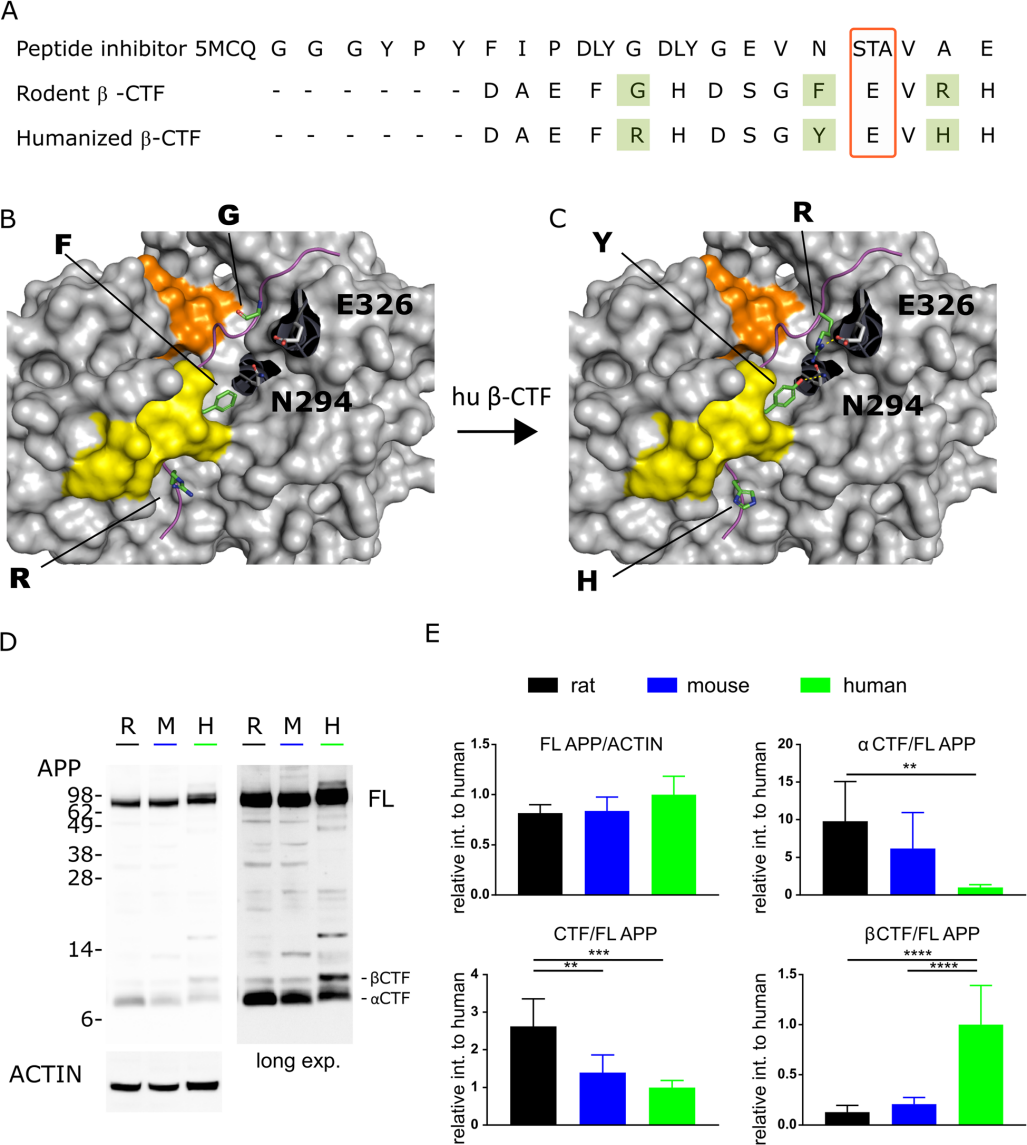
Model of humanized β-CTF bound to BACE1 and western blot analysis of mouse, rat and human APP. **a** Sequence alignment between the peptide inhibitor used in the crystal structure 5MCQ and the humanized β-CTF. Green: the three amino acids different between the human and rodent APP sequence. **b** The BACE structure is presented with its flap [22] in yellow and the 10S loop in orange; a model of rodent Aβ peptide is fitted. **c** Shows the same structure but now modelled with the humanized β-CTF. Two extra interactions between the G676R of the peptide and residue E326 and F681Y and residue N294 of BACE can be noted. **d** Western blot analysis of APP protein in the cerebrum of WT mouse (M), WT rat (R) and human (H), (*n* = 6). The B63 antibody, raised against the C-terminal part of APP, detects both full length APP (FL APP) and C-terminal fragments (CTF, longer exposure). β-ACTIN was used as the loading control. **e** Intensity-based quantification of full length APP, with values normalized to the human sample (*n* = 6, mean ± SD, **p = 0.0063 α-CTF/FL APP, **p = 0.0023 CTF/FL APP, *** *p* = 0.0002, ****p < 0.0001, One-Way ANOVA, Turkey post-hoc test)

We have modelled the interaction of humanized β-CTF with BACE1, using as a template the published crystal structure of BACE1 in combination with an active site peptide inhibitor (PDB ID 5MCQ) [22]. The sequence of the humanized β-CTF was aligned to the peptide in 5MCQ, aligning residue E11 from the humanized β-CTF sequence to the STA query (Threonine) of the crystallized peptide (Fig. 1).

## Results

### Humanized β-CTF provides a better substrate for BACE1

Three amino acid substitutions (G676R, F681Y and R684H) in the Aβ sequence decrease BACE1 processing of rodent APP compared to human APP [14]. We superposed the rodent and humanized β-CTF on a 21 amino acid peptide inhibitor at the binding site of BACE1 (Fig. 1), using the crystal structure PDB id:5MCQ [22]. The modeled binding modes reveal two important extra interactions of the humanized β-CTF with BACE1. G676R (replacing the glycine in the rodent sequence with arginine) provides a large positive charge that interacts with glutamate E326 of BACE1. F681Y introduces an extra -OH group, which acts as a hydrogen bond donor to N294 in BACE1. The two amino acids E326 and N294 of BACE1 require no reorganization or conformational change to make the interactions with the humanized β-CTF (Fig. 1).

We validated the different APP processing in human, mouse and rat brain by western blot analysis (Fig. 1). The ratio of β-CTF over full length APP in human samples is 4.8 (*p* < 0.0001 and 7.8 (p < 0.0001) times higher compared to mouse and rat, respectively, confirming that human APP is a better substrate for BACE1.

### Processing of humanized Aβ APP in the rodent brain

**Fig. 2:**
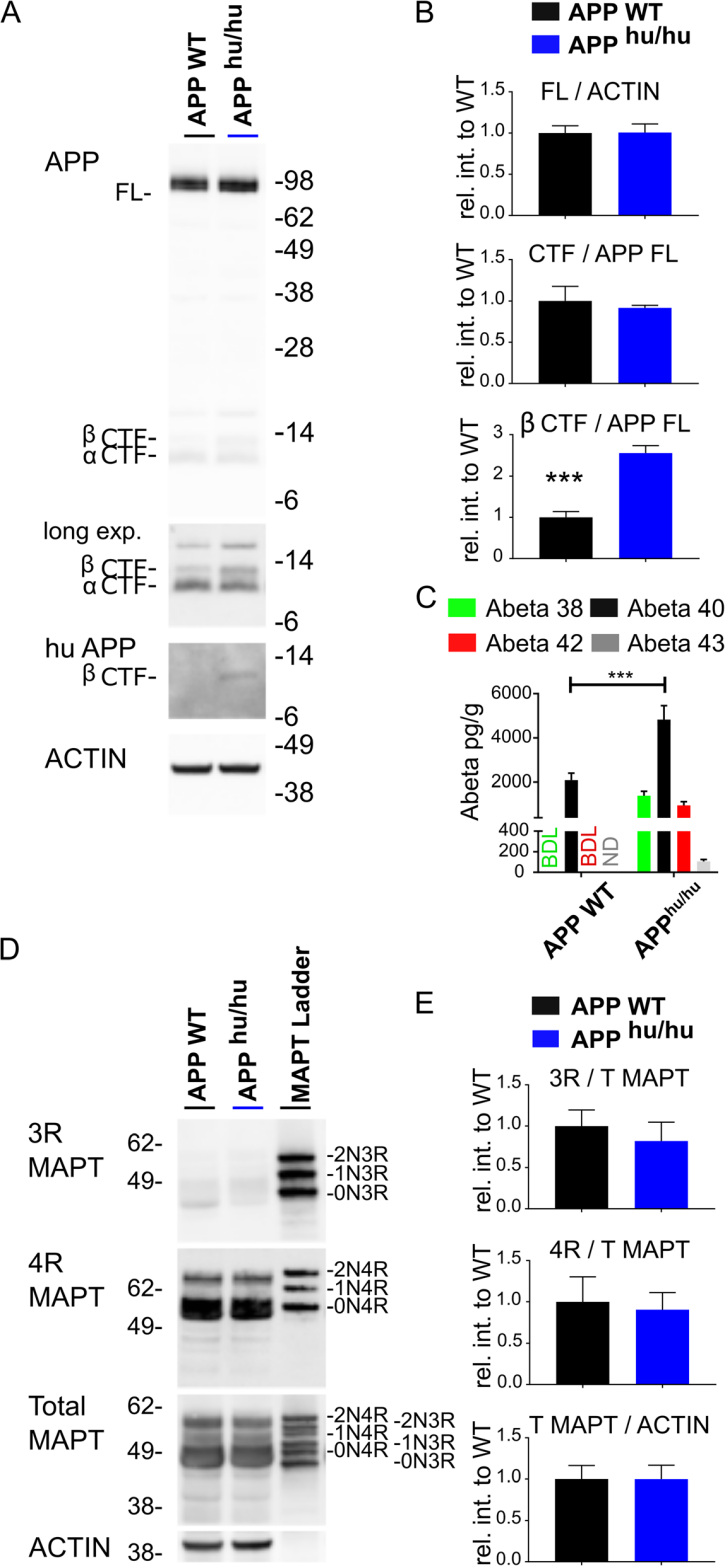
Humanization of the Aβ sequence in mouse affects APP processing. **a** Western blot analysis of APP protein in the cerebrum of 14 weeks old WT and App^hu/hu^ mice (n = 6). B63 antibody detects full length APP (APP FL) and C-terminal fragments (CTF, longer exposure). 82E1 antibody specifically detects human Aβ1-22, and hence the human B-CTF, independently confirming that the mouse *App* gene was successfully humanized. β- ACTIN was used as loading control. **b** Quantification of APP protein in relative intensities (*n* = 6, mean ± SD, *** *p* < 0.0001 two tailed t-test). **c** ELISA analysis of soluble Aβ expressed as pg/g tissue. BD, below detection limit; ND, not determined (*n* = 6, mean ± SD, ***p < 0.0001 two tailed t-test). **d** Immunoblot of total MAPT in mouse cerebrum using the 3Rtau-specific antibody RD3, 4Rtau-specific antibody RD4 and an antibody against total tau. The MAPT ladder shows recombinant human MAPT (0N3R, 0N4R, 1N3R, 1N4R, 2N3R, 2N4R). Notice that mouse MAPT proteins migrate faster than the corresponding human splice variants. β- ACTIN was used as the loading control. **e** Quantification of MAPT relative to WT samples ((*n* = 6, mean ± SD)

**Fig. 3:**
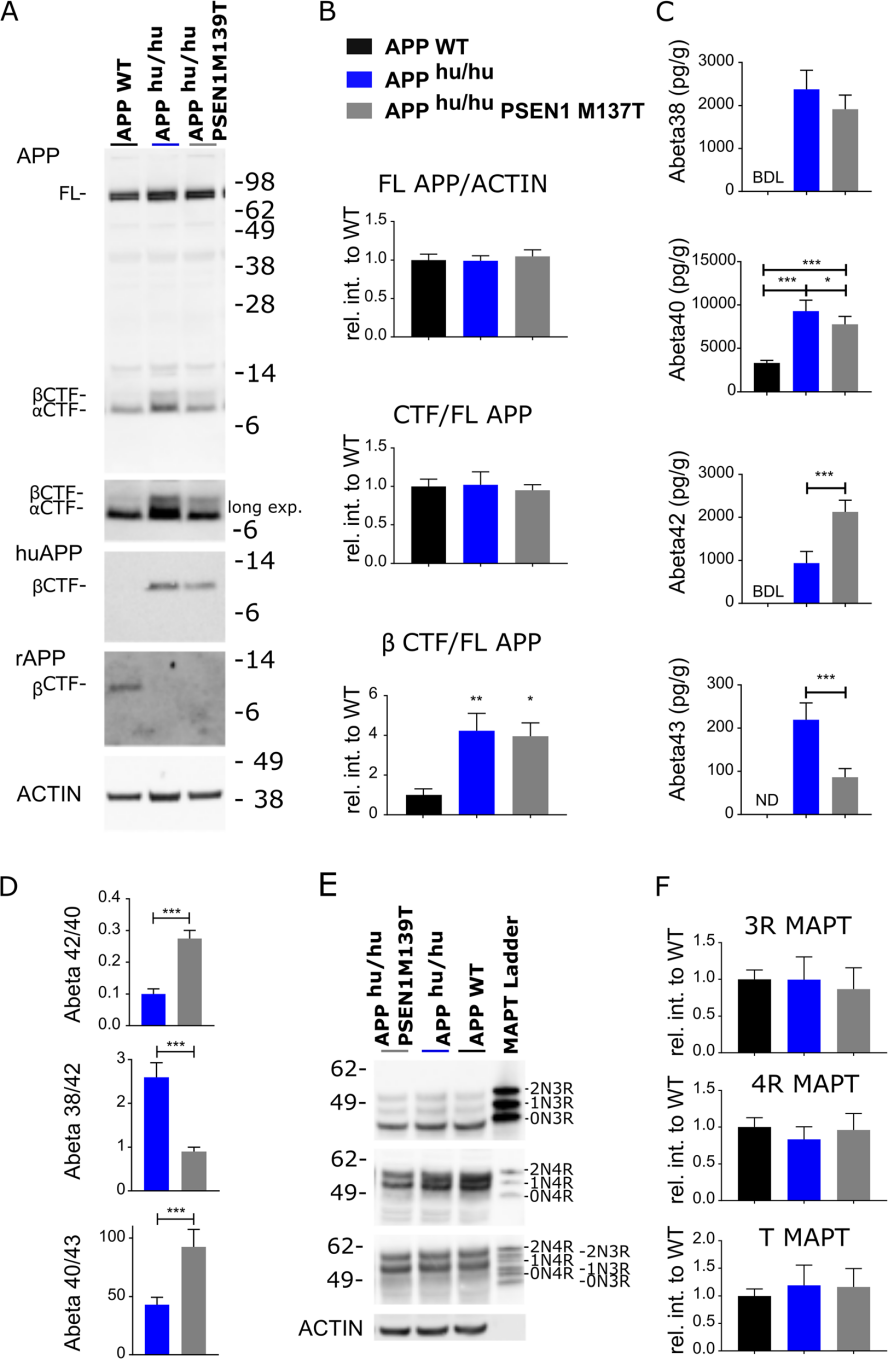
Humanization of the Aβ sequence in rat affects APP processing, while the M139T mutation in PSEN1 results in increased Aβ42 production. **a** Western blot analysis of APP protein in cerebrum of 14 weeks old WT, *App*^hu/hu^ and *App*^hu/hu^;*Psen1*M139T^+/+^ rats (*n* = 6). B63 antibody binds to full length APP (FL APP) and C-terminal fragments (CTF, longer exposure). 82E1 antibody specifically detects human Aβ (1-16), and hence the human B-CTF, independently confirming that the rat App gene was successfully humanized. β- ACTIN was used as a loading control. **b** Quantification of APP protein using relative intensity (*n* = 6, mean ± SD, ** *p* = 0.009, * *p* = 0.017, One-Way ANOVA, Turkey post-hoc test). **c** ELISA analysis of soluble Aβ expressed as pg/g tissue. BD, below detection limit; ND, not determined (*n* = 6, mean ± SD, *** *p* < 0.0001, * *p* = 0.012, One-Way Anova, Turkey post-hoc test). **d** Aβ ratios for App^hu/hu^ and App^hu/hu^;*Psen*1M139T^+/+^ indicate an impairment in γ-secretase cleavage (*n* = 6, mean ± SD, ***p < 0.0001 two tailed t-test). **e** Immunoblot of total MAPT in rat cerebrum with the 3Rtau-specific antibody RD3, the 4Rtau-specific antibody RD4 and an antibody detecting total tau. The MAPT ladder shows recombinant human MAPT (0N3R, 0N4R, 1N3R, 1N4R, 2N3R, 2N4R). Notice that rat MAPT proteins migrate faster than the corresponding human splice variants. β- ACTIN was used as the loading control. **f** Quantification of MAPT relative to WT samples ((*n* = 6, mean ± SD)

We humanized the rodent APP genes (*APP*^hu/hu^, additional files 1 and 2) and analyzed brain homogenates by western blotting (Figs. 2 and 3). Full length APP and total APP-CTF levels were unchanged in humanized rat and mice, but APP-β-CTF signals increased 2.5 fold (*p* < 0.0001) and 4.2 fold (*p* = 0.009), respectively. Conversely, APP-α-CTF signals decreased 0.6 fold (*p* = 0.0067) and 0.7 fold (*p* = 0.0423) in *APP*^hu/hu^ mice and rats. In mice, the Aβ40 levels in brain tissue rose from 2091 ± 130 pg/g in WT to 4827 ± 257 pg/g in the KI model (*p* < 0.0001), while in rats the levels were increased from 3302 ± 1256 pg/g in WT to 9292 ± 516 pg/g in the KI animals (*p* < 0.0001). The higher Aβ40 levels correlate with the fact that APP-β-CTF production in *App*^hu/hu^ rats, and basal Aβ generation in WT rats, are also higher than in the mice (Figs. 2 and 3). Soluble extracted total Aβ measured as the sum of Aβ38 + 40 + 42 raised the amounts measured to 7187 ± 1022 pg/g in the *APP*^hu/hu^ mouse and 12,615 ± 1511 pg/g in the *APP*^hu/hu^ rat. This is approximately half the concentration measured in control human brain, which was 21,141 ± 8712 pg/g.

### M139T mutation in PSEN1 results in a shift in Aβ profile

The M139T FAD mutation [23] alters Aβ38/Aβ42 and Aβ42/Aβ40 ratios without affecting the endopeptidase cleaving activity responsible for release of the APP intracellular fragment [24]. This mutation is predicted not to interfere with Notch signaling [25], which we confirmed as *App*^hu/hu^ rats homozygous for the M139T FAD mutation are fertile and are indistinguishable from their PSEN WT littermates. Brain homogenates of *App*^hu/hu^;*Psen1*M139T^+/+^ mice were analyzed (Fig. 3). The amounts of APP-CTF, APP-β-CTF and total Aβ measured as Aβ38 + 40 + 42 + 43 (11,898 ± 486 pg/g in *App*^hu/hu^ compared to 12,834 ± 676 pg/g in *App*^hu/hu^;*Psen*1M139T^+/+^) are unaffected by the mutation. However, the mutation causes a relative shift towards more Aβ42 production, in parallel with a very small amount of Aβ43 and less Aβ40 (Fig. 3c). This results in an increased Aβ42/Aβ40 ratio (from 0.10 ± 0.01 to 0.27 ± 0.01) and a decreased Aβ38/Aβ42 ratio (2.60 ± 0.33 to 1.48 ± 0.25), indicating less efficient carboxypeptidase-like activity of the γ-secretase. This finding agrees with our previous in vitro work [24]. Unexpectedly, Aβ43 levels were reduced 2.5 times in the brains of homozygous *App*^hu/hu^;*Psen*1M139T^+/+^ rats, resulting in an increased Aβ40/Aβ43 ratio (42.96 ± 6.39 to 92.41 ± 15.02). As total Aβ is unaffected by the mutation, the reduction of Aβ43 is not due to intracellular aggregation, indicating that the M139T mutation shifts APP processing towards the Aβ42 pathway [26]. Disappointingly, the oldest rat we examined (2 years of ages) did not show amyloid plaque pathology. We performed Aβ guanidine extractions on brain homogenates and confirm that there is no accumulation of insoluble Aβ in the mutant rats.

### MAPT expression profile in rat is more complex compared to mouse

One of the reasons to generate a rat model for AD is that rat MAPT is claimed to be more similar to human MAPT, especially at the level of alternative splicing [27, 28]. 3RMAPT is indeed expressed at very low levels in mice (Fig. 2) and is easily detectable in rats. 4RMAPT is abundant in both species and higher mobility bands indicate the presence of 1N4R, 2N4R splice variants (Fig. 3), which become better visible after dephosphorylation (Additional file 3). Rodent MAPT, which is smaller, migrates faster compared to the human MAPT reference ladder. The estimated ratio of 3R to 4R MAPT is 1:13 in the rat brain, and thus very far removed from the 1:1 ratio in human brain. No tangle or plaque formation was observed until the age of 2 years. In single and double KI rats total MAPT and 4RMAPT protein levels are unchanged over time; the expression of the 3RMAPT isoform decreases and the Alzheimer’s disease-relevant AT100 phosphorylation, which is absent at 14 weeks of age in wildtype, single and double KI rats increases with age (Additional file 4). It seems unlikely that the rats will develop amyloid plaque or tangle pathology at a later age.

## Discussion

We created mice and rats harboring a humanized Aβ sequence in the endogenous *App* gene. The models produce about three times more (human) Aβ compared to the WT rodent original strains, but this is still two times less than compared to humans. The rats and mice are suitable controls or starting points to study the effect of transgenes or knock-in mutations on APP processing. An advantage of these new models is that the human Aβ ELISAs routinely used in AD research can be used to study the different Aβ species.

We provide a first example of model utility by introducing the FAD mutation M139T into the endogenous Rat *Psen*1 gene. The PSEN1M139T mutation [20] leads to mean age of disease onset between 39 and 51 years old. Preclinical carriers have relatively high levels of Aβ42 in the cerebrospinal fluid [29]. In vitro this mutant affects the production rates of Aβ38 and Aβ40, while endopeptidase activity is not altered [24]. Our new data confirm these effects, but the strong lowering of Aβ43 and increase in Aβ40/Aβ43 ratio indicate that in vivo the mutation mainly works via a selective shift towards the Aβ42 product pathway and less via destabilization of the enzyme-substrate complex [30].

While our work was ongoing the group of LaFerla generated *App*^tm1.1Aduci^ mice via homologous recombination, introducing the humanized Aβ sequence into the endogenous *App* locus (JAX, Stock No: 032013). Recently, a knock-in rat model was also described by D’Adamio and colleagues [19, 31], which carries a humanized Aβ sequence and a KI of the PSEN1L435F mutation. The L435F mutation affects endopeptidase activity and, therefore, Notch signaling. Surprisingly, while the mutation is embryonically lethal in mice [32], homozygous rats are viable. No explanation for this interspecies difference is currently available [19]. These rats were also reported not to develop amyloid plaques, although it needs to be stated that animals were only followed up to 2 weeks of age.

The fact that our humanized rat model, incorporating a homozygous PSEN mutation, does not develop symptoms of AD at 2 years of age, despite highly pathological Aβ ratios, raises some tantalizing issues in respect to data obtained in the many overexpression models generated over the last 30 years to study AD. The amyloid plaques in these models seem critically dependent on strong overexpression of APP (and sometimes Presenilin). Such overexpression of proteins can lead to many artefacts. The fact that the simple introduction of the PSEN FAD mutations in the rodent homologues does not cause amyloid plaque formation, despite clear alterations in Aβ ratios, suggests that molecular and cellular events upstream and downstream of amyloid plaque formation in humans are lacking in rodents. Aging might be a major contributing effect, as the generation of amyloid plaques in humans spans several decades. Furthermore, it is increasingly recognized that the transition from biochemical accumulation of plaques to disease involves complex feedback loops between glial cells and amyloid plaques, which might be only partially mimicked in rodent brain [33].

## Conclusion

In conclusion, the field should consider using knock-in techniques to introduce mutations or humanize genes when creating next generation models. Furthermore, knock-in of AD mutations in primates, or the use of human stem cells to generate organoids [34], or mouse-human chimeras [35–37], might provide additional ways to dissect the human molecular and cellular aspects of Alzheimer’s disease, moving the field forward.

## Supplementary information

**Additional file 1: Generation of APP KI mice and rats using CRISPR-Cas (A)** The top panel displays an alignment between human Aβ and rodent Aβ peptides. Differences are indicated in red, boxes represent transmembrane domains. The lower panel depicts the genomic organization and sequence (exon 16) for both the mouse and rat *App* genes. Exons are indicated as black boxes, arrows denote primers used for genotyping and sequencing. Underlined sequences were targeted with CRISPR guides; ssODN represent the templates used for homologous recombination. Nucleotides and amino acids indicated in red are the target sequences, nucleotides in green are silent mutations introduced to prevent Cas9 recutting after homologous recombination. **(B)** Sanger sequencing results confirmed the introduction of the point mutations in one strand as shown above the chromatograms. **(C)** PCR analysis of APP KI mice. The digest pattern produced by EcoRI indicates the presence of the KI allele, as the restriction site is destroyed after gene editing.

**Additional file 2: Generation of M139T** ***Psen*1 KI rats by CRISPR-Cas. (A)** Partial sequence of exon 5 of the rat *Psen*1 gene. Underlined sequences indicate the CRISPR guide CRISPR; ssODN represent the template used for homologous recombination. Nucleotides and amino acids indicated in red are the target sequences. **(B)** Sanger sequencing results confirmed the introduction of the point mutations (underlined).

**Additional file 3: Comparing MAPT splice isoforms between mouse and rat.** Analysis of MAPT isoforms after dephosphorylation with alkaline phosphatase (AP). Cerebrum extracts were treated with (+) or without (−) alkaline phosphatase (AP) at 37 °C for 1h and immunoblotted with a 3Rtau-specific antibody RD3, the 4Rtau-specific antibody RD4 and an antibody detecting total MAPT. The middle lane is recombinant human MAPT (ladder). Mice express mainly the 0N4R splice variant compared to the more complex expression pattern in rat brain lysates, which contain all 6 splice forms. The estimated ratio 3R/4R MAPT = 1/13.

**Additional file 4: MAPT protein analysis in two year old rats.** Immunoblot of MAPT in the cerebrum with total tau antibody, 3Rtau-specific antibody RD3, 4Rtau-specific antibody RD4 and phospho-tau antibodies AT270 and AT100 of two year old rats (*n*=2). LE rats* are cerebrum samples from wildtype Long Evans rats aged 14 weeks. MAPT ladder is recombinant human MAPT (0N3R, 0N4R, 1N3R, 1N4R, 2N3R, 2N4R). Notice that mouse MAPT proteins migrating faster than the corresponding human splice variants.

## Data Availability

*App*^hu/hu^ mice and *App*^hu/hu^;*Psen*1M139T rats will be made available upon request.

## Abbreviations

APP: Amyloid precursor protein
Aβ: Amyloid beta peptide
AD: Alzheimer’s disease
WT: Wildtype
CTF: Carboxy terminal fragment
BACE: Beta-site amyloid precursor protein cleaving enzyme
CRISPR: Clustered Regularly Interspaced Short Palindromic Repeats
Hu: Humanized
FAD: Early-onset familial Alzheimer’s disease
PSEN: Presenilin
ELISA: Enzyme linked immunosorbent assays
ssODN: Single-stranded oligodeoxynucleotides
RNP: Ribonucleoprotein
PCR: Polymerase Chain Reaction
MAPT: Microtubule-Associated Protein Tau
sgRNA: Single guide RiboNucleic Acid
KI: Knock-in
PFA: Paraformaldehyde
PBS: Phosphate-buffered saline
BDL: Below detection limit
