# Supplementary figures and images for: Modeling the β-secretase cleavage site and humanizing amyloid-beta precursor protein in rat and mouse to study Alzheimer’s disease
Source: Mol Neurodegener. 2020 Oct 19;15:60. doi: 10.1186/s13024-020-00399-z (PMC7574558; doi:10.1186/s13024-020-00399-z)

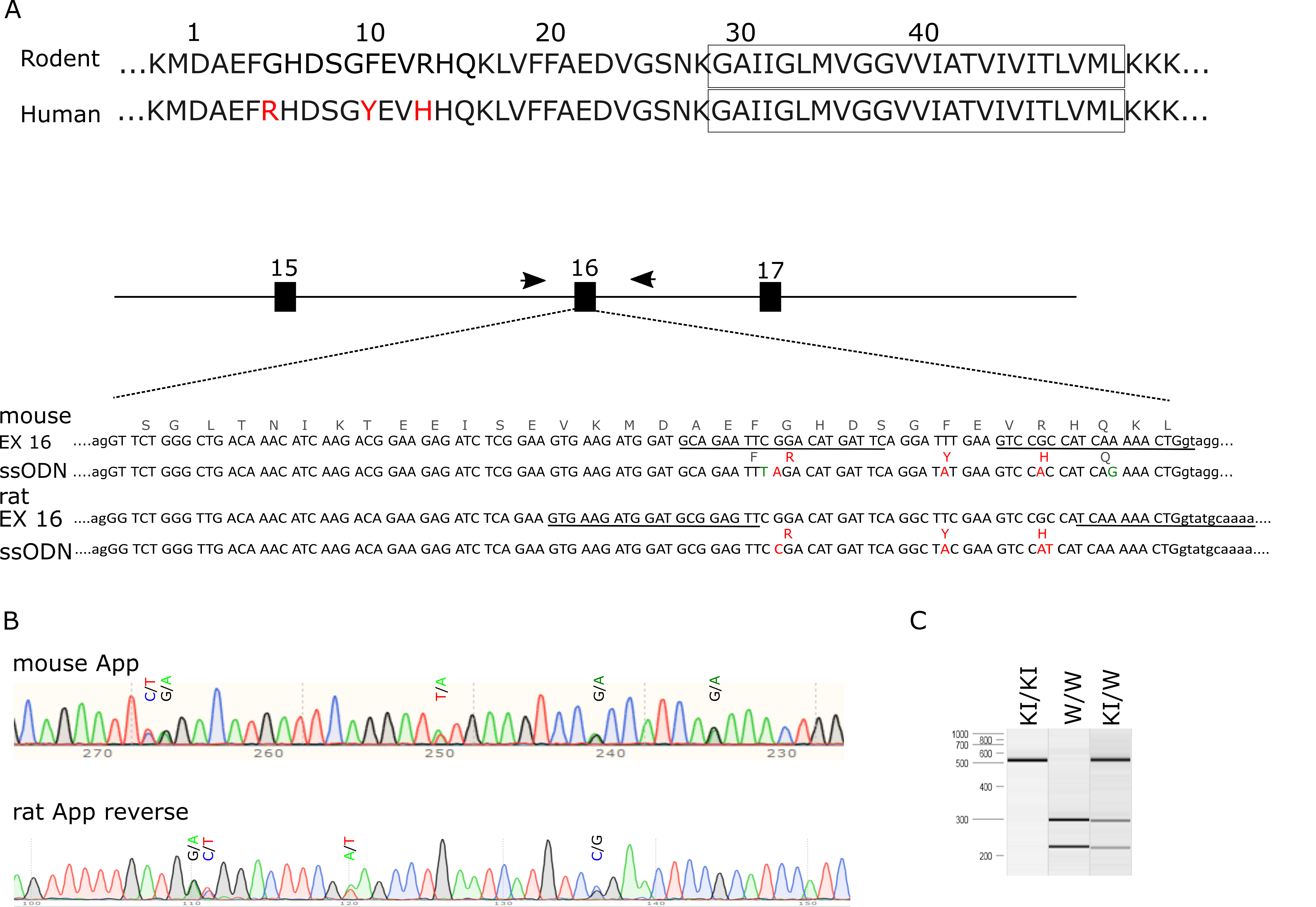

Supplement: Supplementary file 1 — Additional file 1: Generation of APP KI mice and rats using CRISPR-Cas (A) The top panel displays an alignment between human Aβ and rodent Aβ peptides. Differences are indicated in red, boxes represent transmembrane domains. The lower panel depicts the genomic organization and sequence (exon 16) for both the mouse and rat App genes. Exons are indicated as black boxes, arrows denote primers used for genotyping and sequencing. Underlined sequences were targeted with CRISPR guides; ssODN represent the templates used for homologous recombination. Nucleotides and amino acids indicated in red are the target sequences, nucleotides in green are silent mutations introduced to prevent Cas9 recutting after homologous recombination. (B) Sanger sequencing results confirmed the introduction of the point mutations in one strand as shown above the chromatograms. (C) PCR analysis of APP KI mice. The digest pattern produced by EcoRI indicates the presence of the KI allele, as the restriction site is destroyed after gene editing. [file 13024_2020_399_MOESM1_ESM.png]

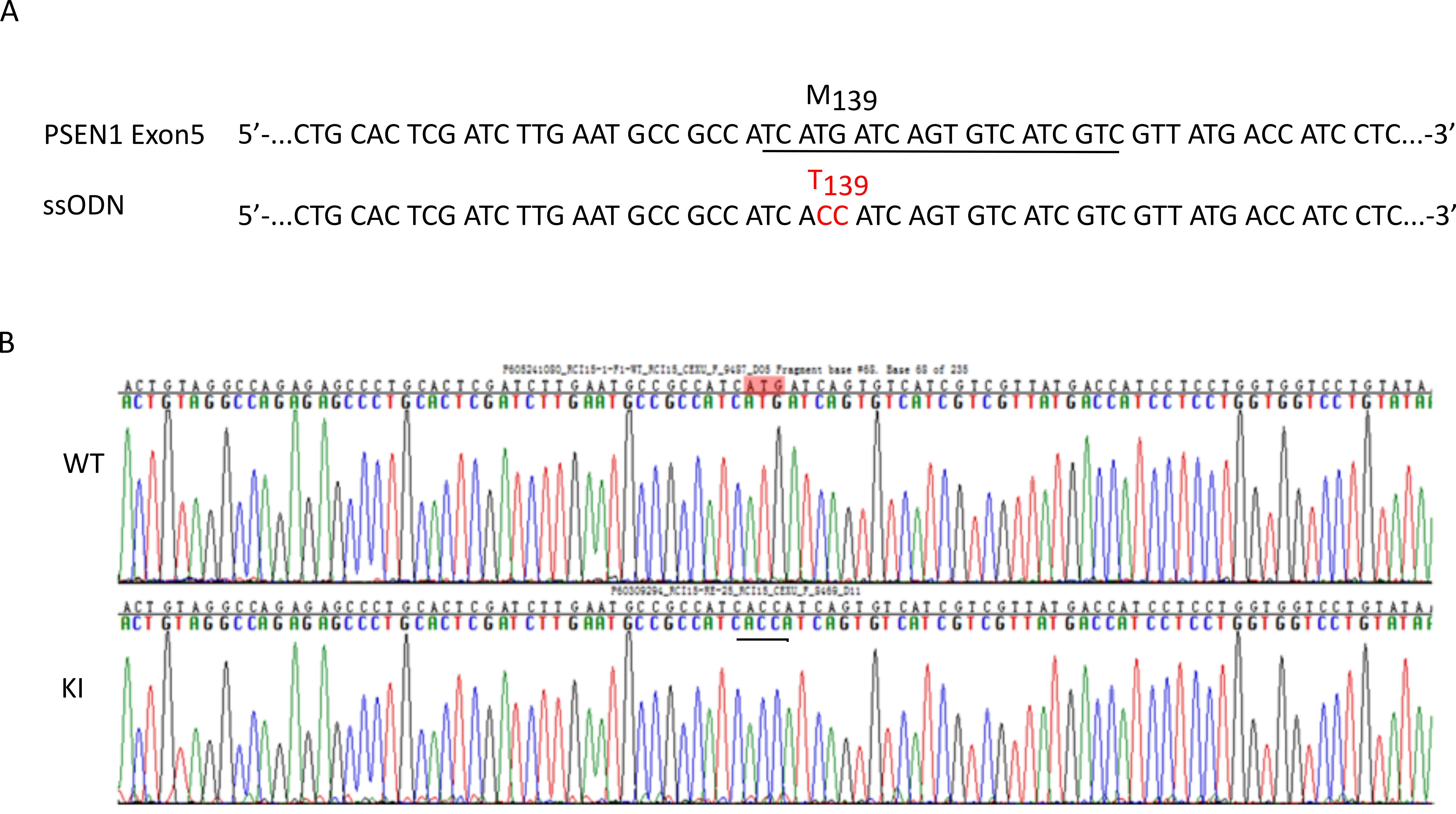

Supplement: Supplementary file 2 — Additional file 2: Generation of M139T Psen1 KI rats by CRISPR-Cas. (A) Partial sequence of exon 5 of the rat Psen1 gene. Underlined sequences indicate the CRISPR guide CRISPR; ssODN represent the template used for homologous recombination. Nucleotides and amino acids indicated in red are the target sequences. (B) Sanger sequencing results confirmed the introduction of the point mutations (underlined). [file 13024_2020_399_MOESM2_ESM.png]

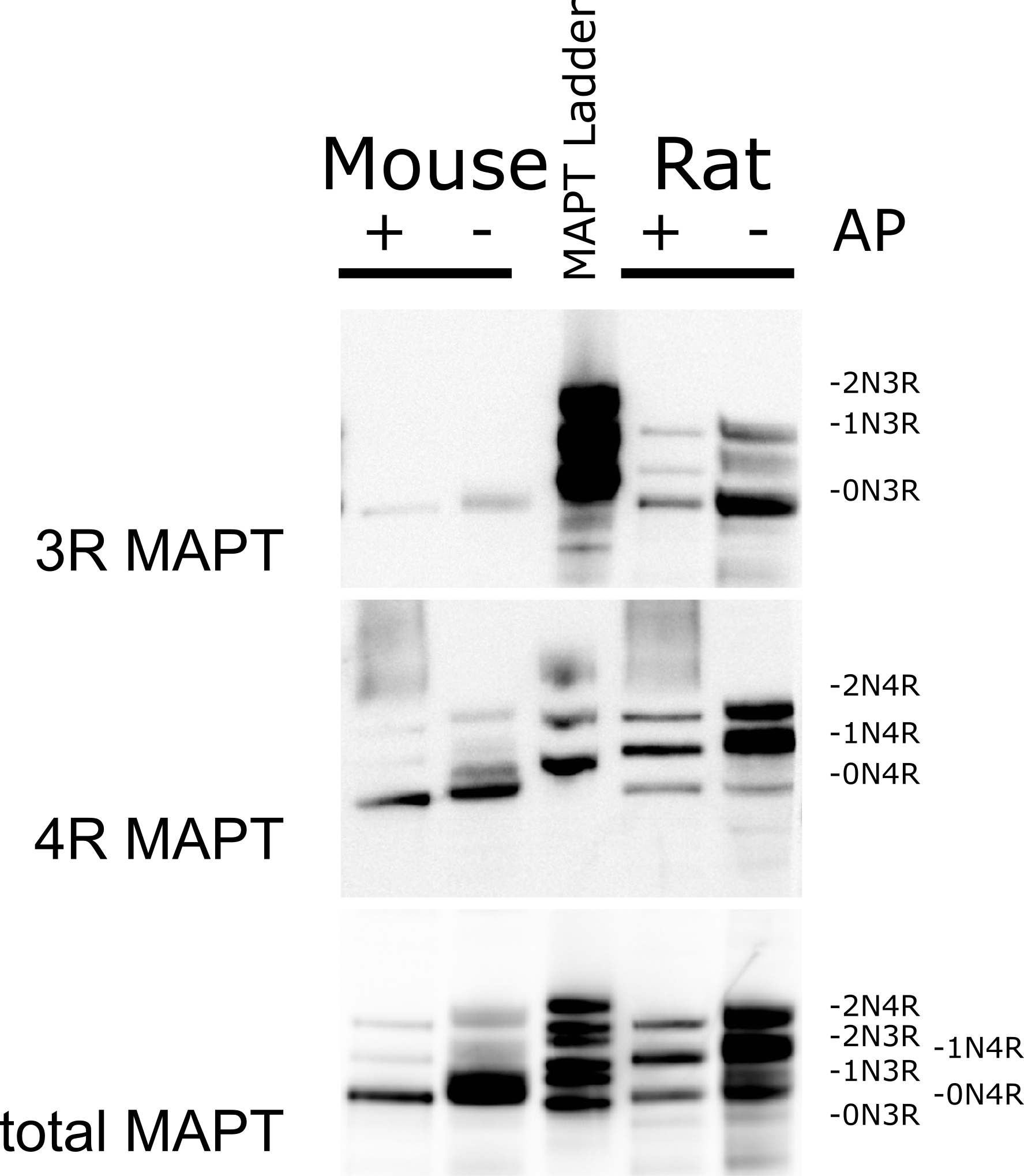

Supplement: Supplementary file 3 — Additional file 3: Comparing MAPT splice isoforms between mouse and rat. Analysis of MAPT isoforms after dephosphorylation with alkaline phosphatase (AP). Cerebrum extracts were treated with (+) or without (−) alkaline phosphatase (AP) at 37 °C for 1h and immunoblotted with a 3Rtau-specific antibody RD3, the 4Rtau-specific antibody RD4 and an antibody detecting total MAPT. The middle lane is recombinant human MAPT (ladder). Mice express mainly the 0N4R splice variant compared to the more complex expression pattern in rat brain lysates, which contain all 6 splice forms. The estimated ratio 3R/4R MAPT = 1/13. [file 13024_2020_399_MOESM3_ESM.png]

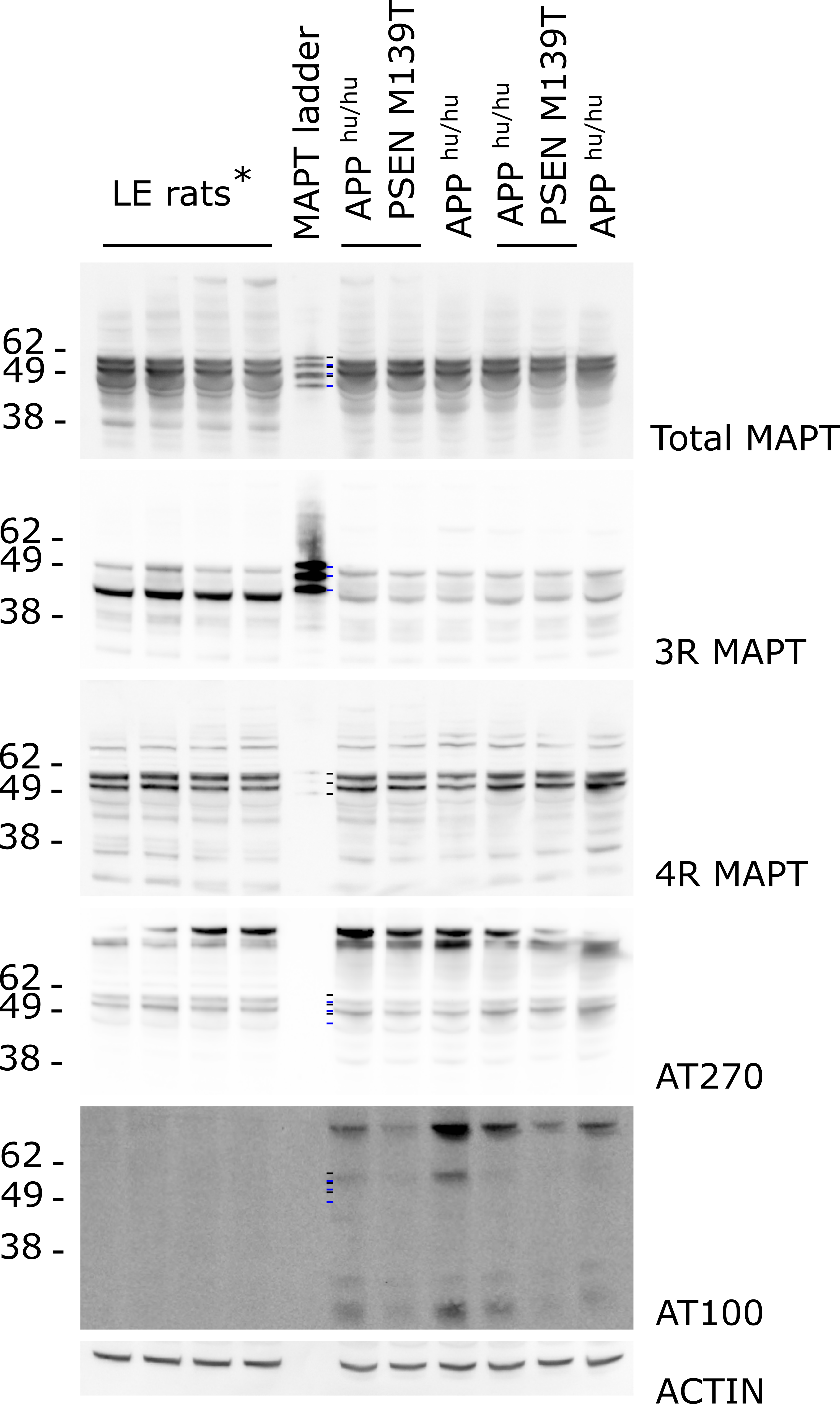

Supplement: Supplementary file 4 — Additional file 4: MAPT protein analysis in two year old rats. Immunoblot of MAPT in the cerebrum with total tau antibody, 3Rtau-specific antibody RD3, 4Rtau-specific antibody RD4 and phospho-tau antibodies AT270 and AT100 of two year old rats (n=2). LE rats* are cerebrum samples from wildtype Long Evans rats aged 14 weeks. MAPT ladder is recombinant human MAPT (0N3R, 0N4R, 1N3R, 1N4R, 2N3R, 2N4R). Notice that mouse MAPT proteins migrating faster than the corresponding human splice variants. [file 13024_2020_399_MOESM4_ESM.png]
